# Experiences and Priorities in Youth and Family Mental Health: Protocol for an Arts-Based Priority-Setting Focus Group Study

**DOI:** 10.2196/50208

**Published:** 2023-11-07

**Authors:** Mandy Archibald, Sharifat Makinde, Nicole Tongol, Sydney Levasseur-Puhach, Leslie Roos

**Affiliations:** 1 University of Manitoba Winnipeg, MB Canada; 2 Children's Hospital Research Institute of Manitoba Winnipeg, MB Canada

**Keywords:** arts-based methods, priority setting, mental health, eHealth, arts-based, art-based, preference, preferences, perspective, perspectives, opinion, opinions, youth, adolescent, adolescents, immigrant, immigrants, native, natives, aboriginal, aboriginals, Indigenous, digital health, telehealth, telemedicine

## Abstract

**Background:**

During the COVID-19 pandemic, eHealth services enabled providers to reach families despite widespread social distancing restrictions. However, their rapid adoption often occurred without community partners’ involvement and without an understanding of how they prioritize aspects of their mental health and associated service provision, both of which promote family and community-centered health care delivery. Establishing priorities in health care is essential for developing meaningful and reliable health services. As such, there is an urgent need to understand how eHealth service users, especially families who may have historically faced oppression and systemic barriers to service access, can best benefit from them. Arts-based approaches can elicit an understanding of priorities by providing an engaging and expressive means of moving beyond readily expressible discursive language and stimulating meaningful dialogue reflective of participants’ lived experiences.

**Objective:**

The purpose of this research is to determine the priorities and preferences of youth; parents or caregivers; newcomers and immigrants; and Indigenous community members regarding the use of eHealth in supporting their mental health using an innovative arts-based priority-setting method.

**Methods:**

This study uses a mixed-methods approach combining qualitative, quantitative, and arts-based research. It follows a survey used to identify key knowledge partners who are interested in improving eHealth services for mental health support in Manitoba, Canada. Knowledge partners interested in group-based priority setting will be contacted to participate. We will facilitate approximately two focus groups across each subgroup of youth, parents or caregivers, newcomers or immigrants, and Indigenous community members using an integrative, quantitatively anchored arts-based method termed the “Circle of Importance” to understand participants’ mental health priorities and how eHealth or technology may support their mental well-being. The Circle of Importance involves placing small objects, whose meaning is determined by participants, on a visual board with concentric circles that correspond to a 5-point Likert scale of importance. Following each focus group, we will evaluate participants’ and focus group facilitators’ experiences of the Circle of Importance using a survey and follow-up structured in-person interviews to garner how we can improve the arts-based approach used in the focus groups.

**Results:**

The PRIME (Partnering for Research Innovation in Mental Health through eHealth Excellence) theme received institutional ethics approval on August 23, 2023. Data collection is projected for August 2023, with follow-up focus groups occurring in early 2024 as required. Data analysis will occur immediately following data collection.

**Conclusions:**

Findings will directly inform a multiyear applied research agenda for PRIME aimed at improving mental health services through engaging key knowledge partners. The results may inform how arts-based methods in a priority setting can reflect aspects of experience beyond the capacities of qualitative or quantitative methods alone, and whether this approach aligns well with a positive experience of research participation.

**International Registered Report Identifier (IRRID):**

PRR1-10.2196/50208

## Introduction

### Background

As the world recovers from the COVID-19 pandemic, health service providers have increasingly turned toward digital technologies to support family mental health. Extensive research documents the disruption that COVID-19 exerted on the lives of families. These disruptions are characterized by challenges across health service access, provider availability, workplace, and home life stress, along with infractions upon coping ability [1-4]. In response to these obstacles, digital technologies, including app-based mental health supports, telehealth, and remote therapy delivery options, helped support in-person service delivery. These digital technologies, henceforth referred to as eHealth, include the provision of mental health support services through digital technology, such as conducting therapy remotely through videoconferencing platforms such as Zoom (Zoom Technologies Inc), Microsoft Teams (Microsoft Corp), or apps.

Although eHealth proved valuable during the COVID-19 pandemic by reducing the strain on health service delivery, its rapid usage made it difficult to adequately develop best practices—and for service providers to have evidence-based guidance—in their absence [5]. The urgency of health system response meant that, in many cases, families and other community knowledge partners were not engaged in determining priorities for and tailoring approaches to maximize the benefits of eHealth services. As the world recovers from the COVID-19 pandemic and in-person services resume, there remains a paucity of literature considering how eHealth services can and should be a part of mental health services for families moving forward. These are essential questions for communities that experience systemic barriers to access related to racial and ethnic oppression, geography-based proximity to care, and mobility challenges due to health status or transportation access. These marginalized communities include Indigenous and newcomer groups in Canada, as well as bedridden or homebound individuals, who benefit from the extension of particular eHealth services such as telehealth [6-9]. This paper presents a protocol for using an arts-based qualitative approach to understanding the family mental health and eHealth priorities and experiences for youth, parents, and caregivers, with particular consideration of the experiences of Indigenous and newcomer communities in the Canadian context.

### Use of eHealth for Mental Health Support

eHealth is a well-accepted component of many mental health services, but during the COVID-19 pandemic, eHealth services were often used as mitigation for an absence of in-person delivery options [10]. eHealth delivery was necessary for reaching individuals in light of quarantine, social distancing, and safety protocols [11-14]. The time-sensitive nature of response mandated by service providers meant that many eHealth services were generated, and many more transformed, to meet public demand [6,8,15,16].

Reviews conducted prior to the emergence of COVID-19 highlight the promise of eHealth in preventing and treating mental health challenges and promoting mental health [15]. For instance, MacKinnon et al [17] found better self-reported mental health and improved anxiety and depressive symptoms for parents of young children, and reduced parenting stress for parents using eHealth interventions. eHealth also shows encouraging results for reducing mental health challenges at the population level, which could help address the adverse effects of the pandemic [15]. The feasibility and acceptability of eHealth are often positively assessed by end users; for example, individuals living with serious mental illness have reported eHealth interventions as feasible and accessible [18]. Additional reviews highlight the promise of eHealth interventions for improving health service delivery; however, further research is needed to identify ways to increase intervention efficacy [16,19,20].

Research conducted during the pandemic was a rapid response to lockdown protocols in the face of increasing need and often without sufficient time to build relationships and ethically engage in best practices with other professionals and knowledge partners. Thus, reviews conducted specifically on the impact of interventions used during the pandemic demonstrate that the lack of legislation, guidelines, and engagement with communities results in a lack of quality of care in some eHealth programs [21-23]. The proliferation of eHealth has outpaced the evidence base, which has functioned as a barrier to policy developments [24] and uptake within particular user groups (eg, older adults) [25], with the strength and quality of evidence being a key characteristic impacting eHealth implementation across contexts [26]. This study context provides a unique and timely opportunity to learn how communities and families want to engage with eHealth moving forward and to identify their priorities for future research and clinical services.

While eHealth services demonstrate promising results for service provision, including reducing commute and wait times, reducing the spread of infections, and improving access to health services [10], understanding how specific groups may benefit from and use eHealth supports is integral to maximizing the benefits for such services. Indeed, eHealth services failed to reach their full potential before the COVID-19 pandemic but have strong potential to enhance health service accessibility [10]. To improve eHealth services, there is a need to understand the experiences of mental health from the individuals and groups who should benefit from them. This understanding is paramount to developing eHealth programs, interventions, and services that align with user priorities.

### Priority Setting and the Benefits of Arts-Based Methods

Priority setting identifies various knowledge user priorities around a topic and, from these, seeks consensus [27]. It is often used to identify and understand health care users’ experiences to inform the health service of health user needs and thus is particularly useful within the context of codevelopment [27]. Priority setting can improve research credibility and practicality for real-life problems and help in developing systematic, user-centered, and applied research programs and associated projects [27]. Within the context of eHealth for mental health services, priority setting can help align eHealth supports with family experiences and needs and inform their improvement and integration within other in-person services, which are sensitive to the users’ local context.

Popular priority-setting methods, such as the James Lind Alliance and the Delphi technique, begin with identifying gaps in the literature, meeting with multiple knowledge users, and voting in multiple rounds before reaching a consensus [27,28]. Consensus is obtained through scoring, ranking, voting, and ordering of items, often achieved through surveys or workshops [27]. Some authors incorporate participatory and playful methods when ranking by using colored sticky notes, cards, and stickers [29,30]. While many of these methods promote empirical ways of knowing, there are opportunities to explore other forms of knowledge that go beyond discursive communication.

Knowledge exists in various forms and can be expressed in a multitude of ways, including more experiential and embodied forms. For instance, children and youth often express themselves using the creative arts, which are regarded as developmentally appropriate in this context [31]. Arts-based methods probe into other forms of knowing—embedded, tacit, emotional, and subconscious—enabling new ways of engaging with experience, new representations, and expressions to emerge, and therefore, new insights to be constructed through and within the relational research space [32]. Despite this knowledge, most priority-setting approaches rely upon discursive knowledge (ie, asking participants to state their priorities and to complete a questionnaire on priorities). On the contrary, arts-based methods provide an opportunity to explore experiences differently and more profoundly and bring to the center what may be missed by traditional research methods. Combining arts-based methods with other narrative methods, such as focus groups, can provide a more holistic understanding of priorities than would otherwise be impossible. Further combining arts-based methods within mixed-methods frameworks can advance complementarity, extend understandings, and provide real-time integration of diverse knowledge forms [33,34]. While arts-based research is proliferating across the health and social sciences, its use in priority settings is nascent. Here, we will engage youth, parents or caregivers, newcomers, and Indigenous community members in arts-based priority setting of their mental health and technology priorities to generate deeper understandings.

### Research Context

The research theme PRIME (Partnering for Innovation in Mental Health through the eHealth Excellence) is the fourth research theme at CHRIM (Children’s Hospital Research Institute of Manitoba). CHRIM is the Canadian Prairie province’s first children’s research facility with the overarching goal of holistically improving the health of children and youth through equitable and innovative research, policies, and interventions [35]. PRIME was developed to address the growing need for mental health services for children, youth, and families that were inflated due to the COVID-19 pandemic [35]. PRIME is developing eHealth models to address youth and children’s mental health needs in Manitoba through equitable partnerships with local communities [35]. The foundational research proposed here directly addresses extant gaps to identify youth mental eHealth needs in Manitoba with broader methodological relevance, and uses and advances the methods for assessing mental health and technology priorities across a variety of knowledge user groups.

## Methods

### Overview

This is a convergent mixed design inclusive of arts-based priority-setting activities and focus groups across the 4 subgroups of youth, parents or caregivers, Indigenous communities, and newcomer and immigrant families [36]. It follows a survey used to identify interested parties and knowledge users in ongoing research engagement in eHealth for mental health in Manitoba, Canada. Arts-based data collection involving the innovative priority-setting approach titled the Circle of Importance will be used to concurrently gather narrative, quantitative, and visual data and will be used as a visual elicitation tool to catalyze focus group interviews. Graphic recording, wherein an illustrator is present during each focus group to visually capture emerging thematic content, will be used as a member checking and dissemination device. This study will be conducted in a large urban capital of a central Canadian province (population 749,607) [37]. We will iteratively collect data at the centrally located CHRIM and analyze the data over a 4-month period across Manitoba.

### Theoretical Framing

#### Arts-Based Priority Setting Using the Circle of Importance

**Figure 1:**
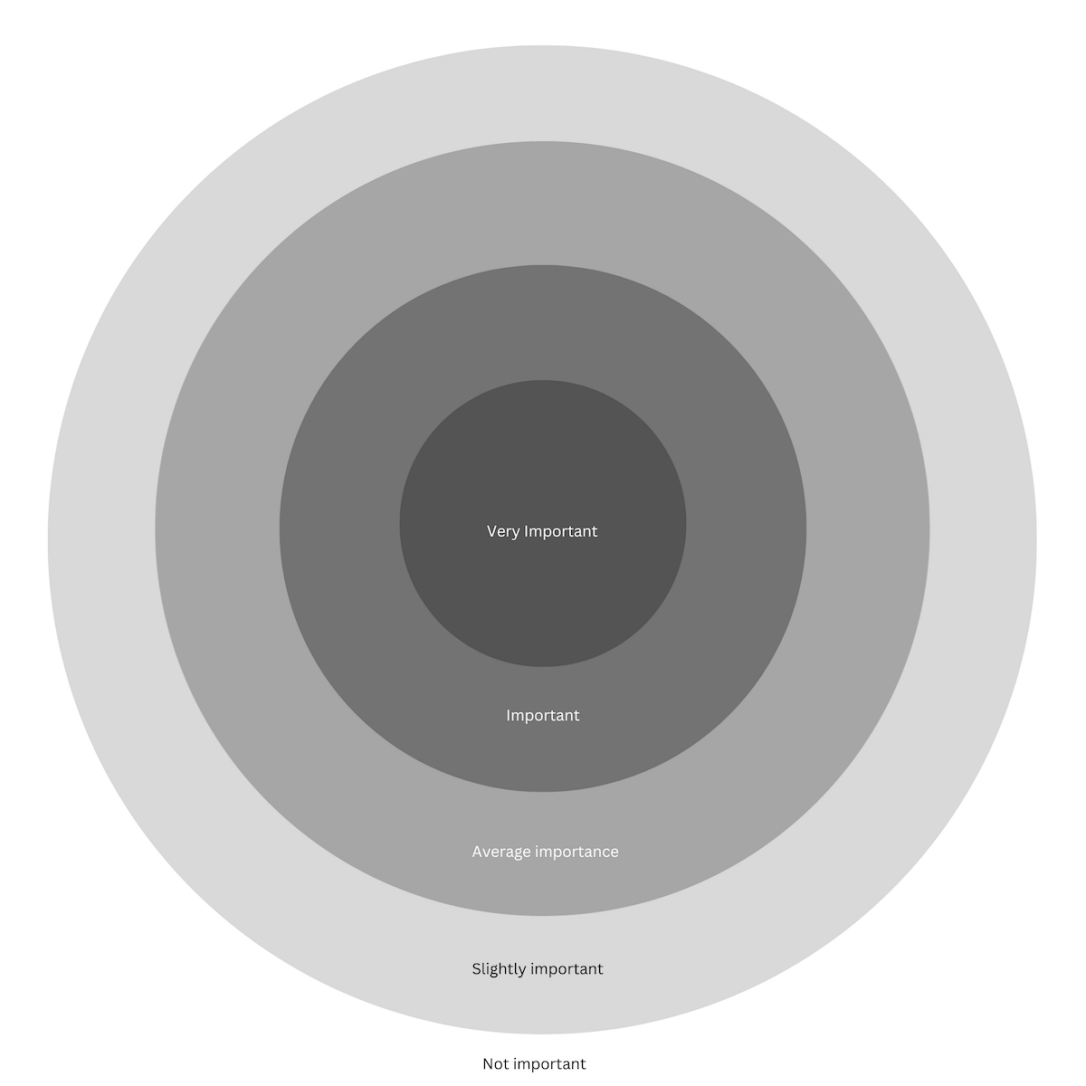
The Circle of Importance, incorporating concentric circles with the embedded Likert scale of importance.

we have developed an innovative arts-based approach to priority setting entitled the Circle of Importance (see Figure 1), integrating aspects of existing priority setting, mixed methods design considerations, arts-based methodologies and theories, and professional experience across arts-based, mixed methods, and qualitative research traditions, building on the first author’s (MA) extensive work in this area [33,36,38-40]. The Circle of Importance draws from a number of methodological traditions and methods applications, including the Center Stage approach. Center Stage involves positioning found objects on a blank board, wherein the center represents the core elements impacting a person’s experience [41]. The participant places other objects of importance in the peripheral areas on the board, with more peripheral placement indicating less significance [42]. As such, center staging encourages participants to reflect and organize their experiences into themes that can be visualized [41].

However, given our specific interest in priority setting and our orientation toward integrating multiple data sources to increase holistic understanding, we adapted the Center Stage process to strengthen the mixed-methods integration potential and mitigate analytic challenges likely to be faced in using the method [39]. Specifically, we extend the blank boards of Overs et al [41] to incorporate 5 concentric circles, which embed a visual 5-point Likert scale on the board for object placement. Participants have the option to select objects provided by the investigative team or objects they have brought themselves and assign meaning to these objects prior to placing them on the board. Each icon or object position is anchored to a quantitative indicator (1-5) aligned with their respective qualitative descriptions (very important to not important) and serves as a visual elicitation stimulus for narrative data attained through the focus group. This approach enables real-time qualitative and quantitative data integration—an area of increasing focus in the mixed methods research context wherein methodological integration and participatory integration methods are garnering interest [34,43]. Here, participants can more readily externalize change in their responses between interview questions.

### Participants and Recruitment

As foundational work to the current proposal, a survey was distributed across Manitoba to identify partners and parties interested in, experiencing, or supporting family mental health. For this study, participants will be purposively recruited from the phase 1 survey. Participants must be aged 16 or older; have experience, interests, or provide support for youth mental health; and identify with 1 of the 4 subgroups (youth, parents or caregivers, newcomer and immigrant families, and Indigenous community groups). These criteria apply to Indigenous and non-Indigenous people with lived experience of mental health and experience as a caregiver or parent of someone experiencing mental health needs. All individuals must reside in Manitoba and be fluent in English. Recruitment will consider age, gender, location, and demographic identity. Five to eight participants will be selected for each subgroup. Youth in this study refers to individuals aged 16-20 years—a definition adopted from the United Nations [44] with modifications made to the upper age category of our sample, recognizing the changing nature of stressors and life stages beyond 20 years of age (eg, increased independence, including place of residence; higher likelihood of employment and postsecondary schooling). The research team will conduct 2 focus group sessions for each subgroup. All participants will receive a CAD $30 per hour (US $22.38) reimbursement through a gift card as a token of appreciation for their participation.

### Data Collection

Focus groups spark discussions among participants and allow them to share similar and dissimilar views [45]. Aside from group restrictions (ie, youth, caregivers, Indigenous community members, and newcomers and immigrants), groups will be heterogeneous in composition to allow diverse perspectives on the topic [45]. Heterogeneity will include age, ethnicity, geographic location, sex, and gender, where appropriate. Furthermore, youth will be subdivided into focus groups reflecting more discrete age ranges (eg, group 1: 15-17 years; group 2: 18-20 years). The lead author has extensive experience in qualitative and arts-based methodologies and will play a key role in conducting the focus groups through facilitation and staff training. A graduate trainee of Indigenous ancestry and with significant qualitative experience will lead the facilitation of the Indigenous community subgroups with instruction and assistance from the lead author. Furthermore, 2-3 facilitators will be present at each focus group to ensure smooth operations and take observational notes of group interactions. Focus groups will be facilitated at CHRIM. A professional graphic recording company will graphically record each focus group to allow for a visually integrative output per subgroup to aid dissemination and member checking.

### Focus Group Approach

#### Overview

**Figure 2:**
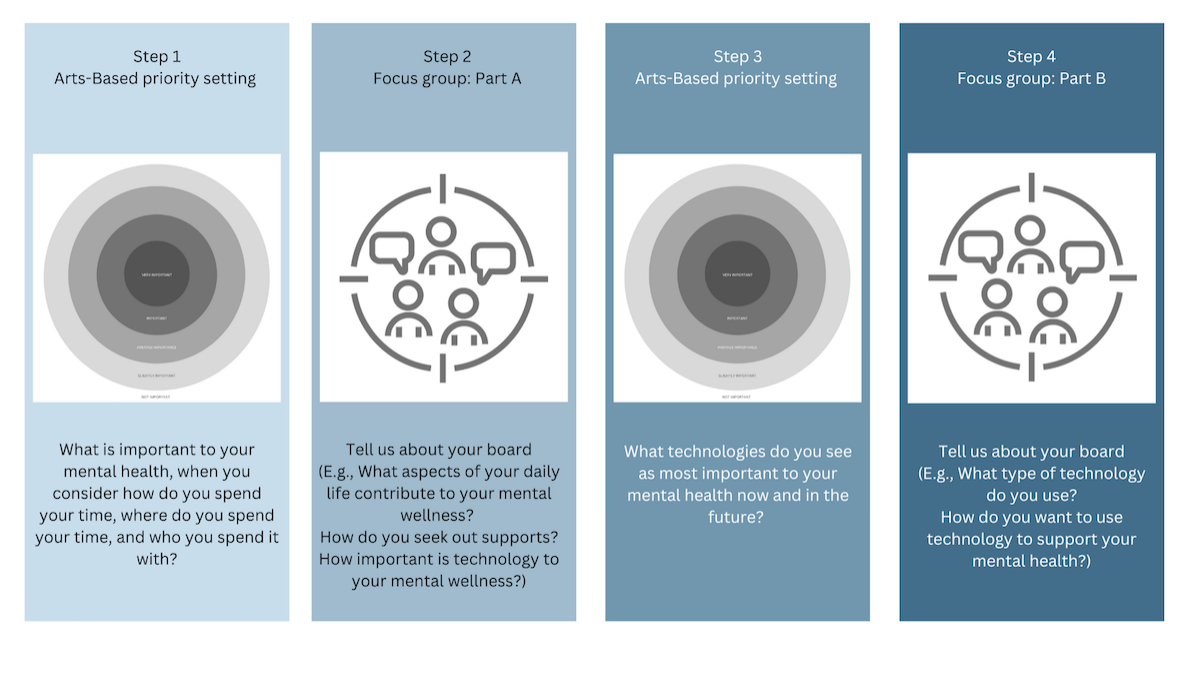
Study sequence.

Focus groups will involve 4 steps, including 2 cycles of the Circle of Importance method followed by semistructured focus group interviews (see Figure 2). Each respective focus group will commence with an icebreaker activity selected based on the specific group. A brief evaluation of participant and facilitator experiences of the Circle of Importance activity will directly follow the completion of each focus group (Multimedia Appendix 1).

#### Step 1: Arts-Based Priority Setting Using the Circle of Importance

The first stage includes identifying what is essential to each person’s mental health using the Circle of Importance (Figure 2). Participants will be instructed to select up to 10 objects that they feel represent an aspect of their life and mental health and directed to position the objects on the Circle of Importance board to reflect what is most important to them. No distinct meaning will be assigned to each object by the facilitators. Instead, participants will be advised to decide what each object represents. Approximately 15 minutes will be provided for each respective Circle of Importance board placement. Facilitators will photograph each board and label the photo with the participant identification number to allow integration with narrative responses throughout the analysis.

#### Step 2: Focus Group Interview—Part A

Following the selection of the items, a facilitator trained in qualitative methods will guide a discussion on the participants’ boards. Two additional facilitators will be present to assist with note-taking of participant nonverbal communication and group interactions and assist with the Circle of Importance method as necessary. The focus group interview will progress from general to specific, beginning with an invitation for participants to describe their board. Participants will also be asked what aspects of their daily life contribute to mental wellness, what contributes to when things are going well, how they seek out support, the importance of technology to their mental wellness, how they use technology, and the importance of technology to receiving mental health services. A simple definition of technology will be provided to participants during the focus group introductions and icebreaker activity.

#### Step 3: Arts-Based Priority Setting Using the Circle of Importance

Subsequent to a break, step 3 will resume with participants reimagining their board in response to the question: What technology do you see as most important to your mental health in the future? Participants will have the option to exchange their icons before partaking in the Circle of Importance method if they so desire. As with step 1, participants will have 15 minutes to complete the Circle of Importance method. At completion, facilitators will photograph each board and label it with the participant ID.

#### Step 4: Focus Group Interview—Part B

After the prioritization method in stage 3, participants will again be invited to discuss their board. The facilitator will first invite participants to describe their board generally, before asking more specific questions about the use and desired use of technology in the context of mental health. Questions will center upon how participants use and want to use technology to support mental health, the types of technologies used, and the technologies regarded as most important to participants’ mental health, now and in the future. Participants will then be asked about the negative impacts of technology use on their own or their family’s mental health, the importance of privacy and confidentiality in the context of technology, preferences around the separation of digital spaces, and perspectives on tracking mental health in app-based technologies. The information gathered during these focus groups will be used to inform the development of a new research theme at the Children’s Hospital Research Institute and associated family and community-centered research and care activities.

### Evaluation of the Circle of Importance

Arts-based approaches to understanding health priorities are an emerging area [46]; as such, feedback from the participants and facilitators on this methodology is crucial for informing its ongoing development. Following the completion of the focus groups, we will evaluate participants’ experiences—including benefits and challenges—of engaging with the Circle of Importance for priority setting. The experiences of facilitators will also be evaluated. We have previously used comparable methods to evaluate methodological innovations, which proved both feasible and useful to methods refinement (eg, Archibald et al [47]). Findings will be used to further refine the Circle of Importance, with sensitivity to the various contexts of its use.

The evaluation will involve descriptive statistics and brief structured follow-up interviews and last approximately 10 minutes per participant. First, participants and facilitators will be provided with a brief questionnaire containing 7 items, structured as a 5-point Likert scale. Questions will center around the feasibility, acceptability, and use of the Circle of Importance for priority setting, as well as challenges and opportunities to improve the method. Following the survey, participants will take part in brief structured interviews containing approximately 6 questions with facilitators to provide an opportunity for additional feedback and to explain the survey responses in a convergent design [45]. Evaluation interviews will occur immediately after the focus group interviews and in the same location.

### Data Analysis

An integrated mixed-methods analytic approach is used in this study. MAXQDA (VERBI GmbH) will be used for data management and to support. Analysis will proceed at the individual and focus group levels. At the individual level, each participant’s artboard, narrative responses, and quantitative Likert response will be analyzed holistically as a case (ie, photos of the participant’s artboard will be analyzed individually as a case and in reference to the focus group context; participants’ Likert scale responses will be recorded in reference to the image on their Circle of Importance, their discussions during the focus group, and in the larger contexts of the focus groups). Inductive thematic analysis will be used. Analysts trained in qualitative and mixed methods will view the photographs of the artboards in tandem while reading the interview transcriptions repeatedly to gain a sense of the whole. Codes will be constructed and applied in accordance with the research questions and assigned descriptions. Case outlines will be provided for each participant and compared within and across subgroups to generate inductive explanatory insights.

With the focus groups, data will be analyzed and organized by subgroups (ie, youth, parents or caregivers, and Indigenous communities). Likert scale responses will be pooled and analyzed descriptively once the objects have been clustered categorically. Thematic labels will be assigned to the objects and reported by prevalence across and within subgroups. Transcripts will be inductively coded; previous transcripts will be reviewed once again when new codes have been identified to ensure comprehensive coding. A variety of strategies, such as joint displays wherein data of various forms are presented in dialogue within a table, will be used to facilitate integration and aid dissemination [48].

The evaluation of the Circle of Importance method will involve data cleaning of the transcribed audio files, and uploading of the transcriptions and survey responses into the mixed-methods software for analysis. Statistical analysis software (SPSS; IBM Corp) will be used to descriptively analyze quantitative data; these will be linked with corresponding narrative explanations of responses and presented using the integrative method of joint displays.

### Ethics Approval

Ethical approval was received from the University of Manitoba Research Ethics Board (HE2023-0139). To protect confidentiality during the focus groups, participants will be asked to keep all information discussed private. Participant quotations will be carefully reviewed to ensure anonymity and to ensure that identifying information is removed.

## Results

Data collection will commence in August 2023. The findings from the study will be used to develop and inform numerous initiatives, including the development of a context-sensitive research theme within the Children’s Hospital Research Institute, a series of publications regarding subgroup level analysis of priorities inclusive of the multiple data sources, and a methodological paper describing the use and evaluation of the Circle of Importance method. Results will be reported at each subgroup level and published approximately in August 2024.

## Discussion

### Priority Setting for eHealth in Mental Health

eHealth has the potential to increase access to health services and improve mental health services for various communities [15]. Every individual experiences mental health differently; therefore, people may have similar or varying values and priorities regarding enhancing their well-being. Similarly, how individuals experience and cope with mental health symptoms varies depending on several factors such as gender, ethnicity, culture, age, access to services, and the intersections of these elements. For example, newcomer and immigrant groups in Canada often face acculturation stressors that affect their mental well-being, and report distress from reporting distress from learning a new language and integrating into a different culture [49]. They experience systemic hurdles to mental health service access, such as language barriers, fear of stigma or discrimination from service providers, and difficulty finding information about accessing needed services [50,51]. Notably, there are different ways of experiencing, defining, and treating mental health and well-being across cultures. For example, spirituality can be a significant cultural factor in one’s experience, with some individuals viewing mental well-being as an inner peace, while others view mental illness as an evil to be expelled [51,52]. Even when born in Canada, children of newcomers and immigrants may experience unique mental health challenges. Studies indicate that they may struggle with a sense of belongingness both in relation to their family’s country of origin and the country they currently reside in [53]. Thus, it is possible that mental health experiences and priorities differ among various groups (eg, parent or caregiver, youth, newcomer, and Indigenous communities). For many Indigenous nations in Canada, well-being is defined holistically, encompassing balance between physical, mental, emotional, and spiritual components of the self. Connections to land, traditional languages, and cultural practices are additionally noted as central to wellness among Indigenous people [54-58]. Wellness also extends beyond the individual, being dependent upon the whole family and community’s well-being through relational considerations [59,60]. The concept of mental illness is a western construct that does not often resonate with traditional ways of knowing, being, and doing nor does it account for systemic and social determinants that influence well-being [61,62]. Therefore, encouraging participants to self-identify priorities and needs aligned with personal and cultural values is critical in promoting safe and respectful practice in research.

Arts-based approaches are well-suited to reflect these unique experiences and priorities for mental health and eHealth that other approaches may fail to identify. Though an emerging methodology, arts-based priority setting has the potential to improve user-centered priority-setting research. Literature highlights popular prioritization methods such as the James Lind Alliance, the Delphi process, and the Nominal Group Technique for involving end users and other knowledge users [63,64]; however, these techniques demonstrate higher ontological and epistemological rigidity than creatively situated approaches. Arts-based methods, for instance, stimulate critical knowledge about human experience from the unconscious mind while provoking transformation [46]. Because of their ability to encourage authentic participation and elicit new and deeper responses to experiential questions, arts-based approaches to priority setting offer unique potential in discovering meaningful experiences and mental health and eHealth priorities across diverse subgroups [32]. There is often a lack of clarity in evaluating arts-based methods in research and priority-setting contexts [46]. This study addresses this challenge using a 5-point Likert scale with the Circle of Importance, quantifying priorities while remaining flexible enough to qualitatively convey participant priorities and experiences. Furthermore, using surveys and semistructured interviews in this study permits the evaluation of the effectiveness of arts-based methods for priority setting while enabling future tailoring of the approach to various subgroups commonly engaged in priority setting.

### Limitations

The Circle of Importance method is a novel innovation, and while we will evaluate its use and acceptability, the activity may not go as planned and may not produce the anticipated results. However, related research suggests that participants will respond well to this form of visual elicitation and arts-based methodology [41,65]. Additionally, there may be some limitations in recruitment. Despite our best efforts, we may not be able to recruit equal numbers across the 4 subgroups.

### Future Directions

While data collection has not yet commenced, we recommend that researchers consider the use of arts-based approaches in trying to capture complex and varying experiences among individuals. It is also important to try and be aware of the different factors (eg, ethnicity, age, and the intersection of multiple identities) that can contribute to individuals’ experiences of their mental health and well-being. Thus, collecting data from various groups with differing identities can provide a richer and fuller picture of their experiences.

## Appendix

Multimedia Appendix 1 Circle of Importance and participant evaluation questions.

## Abbreviations

CHRIM: Children’s Hospital Research Institute of Manitoba
PRIME: Partnering for Innovation in Mental Health through the eHealth Excellence

## Data Availability

The data sets generated and analyzed during this study are not publicly available due to the identifying nature of the data. Data cannot be made public in order to protect participant confidentiality and privacy. However, deidentified data sets are available from the corresponding author upon reasonable request.
