# Supplementary material for: Experiences and Priorities in Youth and Family Mental Health: Protocol for an Arts-Based Priority-Setting Focus Group Study
Source: JMIR Res Protoc. 2023 Nov 7;12:e50208. doi: 10.2196/50208 (PMC10664011; doi:10.2196/50208)
Supplement: Multimedia Appendix 1 [file resprot_v12i1e50208_app1.pdf]

Post Circle of Importance Facilitator Questions

For each of these statements below, circle the response that characterizes how you feel:

1 = Strongly Disagree, 2 = Disagree, 3 = Neither Agree nor Disagree, 4 = Agree, 5 = Strongly Agree

	Strongly Disagree	Disagree	Neither Agree nor Disagree	Agree	Strongly Agree
I felt that **participants understood** the Circle of Importance method after my explanation	1	2	3	4	5
Participants required **minimal assistance** in **selecting their objects** for the Circle of Importance method	1	2	3	4	5
Participants required **minimum assistance** with **creating their artboards** for the Circle of Importance method .	1	2	3	4	5
There was **enough time** for participants to complete their artboards for the Circle of Importance method.	1	2	3	4	5
The artboards **effectively communicated** the participant's experiences and priorities.	1	2	3	4	5
The artboards helped participants **verbally express** their experiences and priorities effectively	1	2	3	4	5
I think that **participants enjoyed** the Circle of Importance method.	1	2	3	4	5
I felt **satisfied overall** with **facilitating** the Circle of Importance method.	1	2	3	4	5

Post Circle of Importance Facilitator Interview Questions

1. Please describe your experience facilitating the Circle of Importance method.

2. What, if any, benefits did you observe when facilitating the Circle of Importance method? (e.g., benefits to participant expression or reflection)

3. To what extent do you think participants were able to understand the instructions for the Circle of Importance method? How, if at all, could the instructions have been improved?

4. How would you describe participants' responses to this method? (e.g., prompts: did they seem comfortable/uncomfortable? Did they enjoy it/not enjoy it?)

5. What, if any, challenges did you encounter when facilitating the Circle of Importance method?

6. What specifically about the Circle of Importance method do you think was helpful and worked well for participants, if anything?

7. Are there any areas that the facilitation of this method could be improved?

Post Circle of Importance Participant Questions

For each of these statements below, circle the response that characterizes how you feel:

1 = Strongly Disagree, 2 = Disagree, 3 = Neither Agree nor Disagree, 4 = Agree, 5 = Strongly Agree

	Strongly Disagree	Disagree	Neither Agree nor Disagree	Agree	Strongly Agree
The Circle of Importance method helped me **reflect** on my experiences and priorities with mental health.	1	2	3	4	5
I found the Circle of Important method useful to **expressing** my experiences and priorities.	1	2	3	4	5
I **enjoyed** the exercise of arranging my important objects on the board.	1	2	3	4	5
I found the Circle of Importance method **easy** to carry out.	1	2	3	4	5
Enough **time** was provided for the Circle of Importance method.	1	2	3	4	5
The items and icons provided for the Circle of Importance method were **appropriate**.	1	2	3	4	5
I felt like the **facilitators were understanding** of my explanation of what the objects on my board represented.	1	2	3	4	5
I would **recommend** the Circle of Importance method as a way of expressing priorities and experiences for research	1	2	3	4	5

Post Circle of Importance Participant Interview Questions

1. What were your experiences like participating in the Circle of Importance method?

2. What, if anything, did you find useful about the Circle of Importance method?
(prompts: Did the method help reflect your experiences and priorities? Did the method help express yourself?)

3. Did the Circle of Importance method bring up any emotions for you, and if so, which emotions?

4. If you've participated in interviews or focus groups in the past, how did this method compare?

5. What if any changes would you like to see to the selection and number of objects provided for the Circle of Importance method?

6. What, if anything, could be done to improve the Circle of Importance method? (e.g., duration allotted for method; clarity of instructions?).
